# Getting the basics right-staging in head and neck cancer

**DOI:** 10.1186/1758-3284-1-16

**Published:** 2009-06-09

**Authors:** Nimesh Narendra Patel, Callum Faris, Tahwinder Upile

**Affiliations:** 1 ENT Department, Southampton University Hospitals, Southampton, Hampshire, UK

## Abstract

The cornerstone of oncology literature and therefore medical practice is the ability to compare outcomes of treatment modalities for different stages of cancer.

The head and neck represents a complicated anatomical region with multiple tumour sites and sub-sites. Modern management necessitates accurate staging to allow meaningful discussion between all members of the multidisciplinary team. Variations in techniques utilized for staging laryngeal cancer may lead to inconsistencies in staging and result in inaccuracies in interpreting comparative interventional studies.

We call for standardisation in assessment of head and neck cancers in order to maximize the accuracy of clinical staging. We suggest a schema that we currently use in our institution for staging of all head and neck cancers and a schema for upper aero-digestive tract endoscopy.

## Introduction

The cornerstone of oncology literature and therefore medical practice is the ability to compare outcomes of treatment modalities for different stages of cancer.

The head and neck represents a complicated anatomical region with multiple tumour sites and subsites. Modern management necessitates accurate staging to allow meaningful discussion between all members of the multidisciplinary team. Organ preservation treatments (especially surgery) demand accurate tumour mapping to determine feasibility.

## Discussion

Most published papers now adhere to the International Union Against Cancer (UICC) or the American Joint Committee on Cancer (AJCC) in staging method and result reporting. However, the literature rarely details the techniques used to stage the tumour. Surgical en bloc resection would be expected to provide definitive tumour staging; however, surgical specimens are subject to shrinkage artefact and errors of orientation. Recent trans-oral laser microsurgical techniques can also provide difficulties with pathological staging due to the piecemeal resection procedure and resultant multiple specimens generated. Staging of tumours treated with non-surgical techniques; do not have the benefit of cross-referencing clinical staging with pathological staging.

Staging by imaging is subject to inaccuracies, being relatively insensitive to superficially spreading upper aerodigestive tract mucosal tumours [1]. CT scanning is also thought to under-stage diseases in some subsites and MRI over-stage tumours in some subsites [2]. The traditional panendoscopy provided detailed visual and palpation evidence of tumour extent. When coupled with awake fibre-optic laryngopharyngosopy, dynamic assessment was added – essential in staging laryngeal disease. There is even evidence to suggest that videostroboscopy has a role to play in tumour staging [3]. The recent move towards fibre-optic evaluation only, might risk losing some of the detailed information gained by a full panendoscopy under general anaesthesia [4], although detailed comparative studied have not been performed to answer this question. Two modern additions to the panenedoscopy- use of the operating microscope and the Hopkin's rod rigid telescope system (with straight and angled lenses) have been shown to change staging [5,6].

In our institution we use the schema in Appendix 1 for staging head and neck cancers. The panendoscopy has the elements described in Appendix 2. Imaging and pathological reporting is performed to the recommendations of the Scottish Intercollegiate Guidelines Network (SIGN) guidelines on the diagnosis and management of head and neck cancer [7].

## Conclusion

We call for standardisation in assessment of head and neck cancers in order to maximize the accuracy of clinical staging, a key recommendation in the UK National Head and Neck Cancer Audit [8]. Only once this most fundamental part of head and neck cancer management is achieved can we meaningfully interpret patient outcomes from different treatment modalities and undertake informative comparative interventional studies.

## Competing interests

The authors declare that they have no competing interests.

## Authors' contributions

NNP and TU contributed to conception and design and were involved in drafting the manuscript. CF revised the manuscript critically for important intellectual content. All authors read and approved the final manuscript.

## Appendix 1

### Schema for diagnostic work up of a head and neck cancer patient

Full ENT history and Exam

Outpatient naso-laryngoscopy with photo/video documentation

Videostroboscopy (larynx) with photo/video documentation

CT scanning – head/neck/chest

MRI scanning/PET-CT/USS/FNAC, as indicated, pre-biopsy (if possible)

Laryngopharyngoscopy and direct bronchoscopy and oesphagoscopy with 0° and 30/90° endoscopes with photo/video documentation

## Appendix 2

### Schema for panendoscopy

Palpation of neck

Examination of oral cavity

Pharyngoscopy of all subsites

Laryngoscopy (with Pilling's Laryngoscope), bimanual palpation

Suspension Laryngoscopy (Pilling's Laryngoscope), examination of larynx with microscope and 0° Hopkins rod, then re-examine laryngeal ventricles, anterior commissure and sub-glottis with ridged 30° Hopkins rod (including photo documentation). Palpation of larynx with microinstruments

Oesphagoscopy-rigid/flexible and Rigid Bronchoscopy
